# Comparative analysis of antibiotic resistance genes between fresh pig manure and composted pig manure in winter, China

**DOI:** 10.1371/journal.pone.0317827

**Published:** 2025-01-29

**Authors:** Shuai Huang, Minghui Xing, Haifeng Wang

**Affiliations:** 1 School of Environmental Engineering, Yellow River Conservancy Technical Institute, Kaifeng Key Laboratory of Food Composition and Quality Assessment, Kaifeng, China; 2 School of Life Sciences, Henan University, Kaifeng, China; Kampala International University - Western Campus, UGANDA

## Abstract

Antibiotic resistance is a critical global public health issue. The gut microbiome acts as a reservoir for numerous antibiotic resistance genes (ARGs), which influence both existing and future microbial populations within a community or ecosystem. However, the differences in ARG expression between fresh and composted feces remain poorly understood. In this study, we collected eight samples from a farm in Kaifeng City, China, comprising both fresh and composted pig manure. Using a high-throughput quantitative PCR array, we analyzed differences in ARG expression between these two types of manure. Our findings revealed significant differences in ARG profiles, as demonstrated by principal coordinate analysis (PCoA). Further analysis identified 39 ARGs (log2FC > 1, *p* < 0.05) in composted pig manure, with 25 genes downregulated and 14 upregulated. Notably, *tetB-01*, *blaOCH*, and *blaOXY* were the most abundant in composted pig manure compared to fresh manure. Additionally, 16S rRNA species profiling revealed that the composting process significantly altered the microbial community structure, with an increased abundance of Firmicutes and a decreased abundance of Bacteroidetes in composted pig manure. In summary, composting substantially transforms both the microbial community structure and the ARG profile in pig manure, underscoring its potential role in modulating the dynamics of ARGs in agricultural environments.

## Introduction

Antibiotics, the cornerstone of human and veterinary medicine for combating bacterial infections, have become pervasive in diverse environments worldwide [1]. This widespread use and contamination of antibiotics have accelerated the proliferation of antibiotic resistance genes (ARGs), posing a growing threat to public health [2]. The misuse or overuse of antibiotics in both clinical and agricultural settings exacerbates this problem, particularly by promoting the enrichment of ARGs within the intestinal microbiota of animals [3,4]. A significant proportion of antibiotics administered to humans and livestock remains unmetabolized and is subsequently released into the environment through multiple pathways. These include direct manure application, leaching into soil, surface runoff, and even atmospheric dispersion [5,6]. Such environmental contamination facilitates the dissemination of ARGs beyond their original sources. Of particular concern is the role of mobile genetic elements, such as plasmids and integrons, which act as vectors for horizontal gene transfer. These elements enable ARGs to spread among microbial populations, infiltrate agricultural crops, and ultimately enter the human body through the food chain [7]. This dynamic amplifies the risks to public health by increasing exposure to resistant bacteria. Moreover, the emergence of multidrug-resistant “superbugs,” particularly within livestock production systems, highlights the critical urgency of addressing bacterial resistance in agricultural environments [8,9]. These superbugs pose a significant challenge to current antibiotic therapies, underscoring the need for comprehensive strategies to mitigate ARG proliferation and its associated risks.

Composting, a sustainable method for aerobic degradation of organic waste, is increasingly utilized to manage livestock manure, transforming it into valuable organic fertilizers [10]. This process naturally generates heat and involves dynamic changes in temperature, pH, oxygen, moisture, and nutrient levels, creating diverse microenvironments that support various microbial populations [11]. Research has demonstrated that composting can effectively break down antibiotics and reduce the abundance ofARGs, with most studies focusing on ARGs associated with tetracycline and sulfonamide resistance [12–14]. The composting process unfolds through four distinct phases: mesophilic, thermophilic, cooling, and maturing, each marked by specific temperature profiles driven by microbial activity. In-depth investigations into these phases have employed metagenomic and time-series metatranscriptomic analyses to monitor the evolving microbial communities. Findings from these studies highlight that composting significantly lowers the overall expression levels of the manure resistome. This resistome predominantly includes genes that confer resistance to a broad spectrum of antibiotics, such as tetracycline, vancomycin, fluoroquinolones, β-lactams, aminoglycosides, and efflux pumps [14]. These insights underline the effectiveness of composting not only in nutrient recycling but also in mitigating the spread of antimicrobial resistance within agricultural settings.

Feces are a significant source of antibiotic pollution, particularly in China, where an estimated 618 billion kilograms of pig manure are produced annually [15]. The absorption of veterinary antibiotics by animals is often incomplete, resulting in their excretion and subsequent use as fertilizer, which introduces these compounds into the soil and promotes nutrient recycling [16]. The widespread practice of adding subtherapeutic levels of antibiotics to animal feed not only enhances growth but also promotes the proliferation of antibiotic resistance traits in feces, amended soils, and aquatic environments such as rivers and sediments [17–20]. Furthermore, the inclusion of metals in pig feed as growth promoters and disease prevention agents contributes to the long-term co-selective pressure for antibiotic resistance [21]. The extensive scale of livestock farming and antibiotic usage in China offers a unique lens through which to assess the environmental impact of large-scale agricultural practices on ARGs. Previous studies have established a direct link between the presence of *tetracycline resistance* (*tet*) genes in soils proximal to pig feedlots and the levels of tetracycline residues found in these areas [22]. This correlation invites further investigation into the diversity and abundance of ARGs beyond just tet genes, underscoring the need for a comprehensive understanding of the pathways through which antibiotics and ARGs enter and affect ecosystems.

Recent advances in research are intensifying the focus on the presence of ARGs in livestock waste, a major environmental and public health concern. High-throughput sequencing technologies have revolutionized our ability to analyze the diversity and abundance of hundreds of resistance genes across environmental samples, revealing that livestock and poultry manure are significant reservoirs of ARGs [23–25]. These findings underscore the public health implications of ARGs, which, through environmental dissemination, can influence selection pressures on bacterial communities and increase the prevalence of resistance [26,27]. Even commensal bacteria, generally harmless, can act as vectors for resistance genes, potentially transferring them to pathogenic bacteria.

To investigate ARGs, researchers have developed both conventional culture-based methods and advanced molecular techniques, including PCR-based assays which offer simplicity and precision in detecting resistance genes [28,29]. High-throughput quantitative PCR (HT-qPCR) and deep sequencing further enhance the capability for broad-spectrum ARG detection and quantification in complex biological matrices. This study leverages PCR arrays to assess and compare the expression levels of ARGs in fresh versus composted pig feces, aiming to explore how the composting process affects the dynamics of antibiotic resistance gene expression. This approach provides critical insights into the persistence and transformation of ARGs during manure management practices.

## Materials and methods

### Sample collection

In February 2024, we collected four samples of fresh pig manure from a farm in Kaifeng City, Henan Province, China. These samples were designated as the control group and labeled A1, A2, A3, and A4, respectively. After one week, we collected four samples of composted pig manure, which had been composted in the open air for one week at the same farm. These samples were part of the experimental group and were named B5, B6, B7, and B8, respectively. Previous studies have documented both short-term and long-term composting processes [14]. At this particular farm, the composting process is a passive, open-air system without manual intervention. Due to variations in operational practices, many farms only compost manure outdoors and subsequently transfer it to septic tanks; thus, the farm does not monitor or record temperature and humidity levels during composting, nor is the compost manually turned or otherwise manipulated.

The daily feces are collected, pooled, and then subjected to composting and fermentation. Standard farm practices for medication and feed management are maintained as per usual protocols. The farm has been operating for over five years and exclusively uses veterinary drugs from the same supplier, administered according to the manufacturer’s instructions. Manure samples were collected using a sterile manure sampling tube, immediately stored in an ice box, transported to the laboratory within 12 hours, and stored at −80 °C until DNA extraction.

### Isolation and extraction of bacterial genomic DNA from pig manure

Genomic DNA was extracted from the pig manure samples using the SPINeasy DNA Kit (MP Biomedicals, California, USA) following the manufacturer’s instructions. The quality of the extracted DNA was verified by electrophoresis on a 1% agarose gel. DNA concentration and purity were assessed using a NanoDrop 2000 UV-vis spectrophotometer (Thermo Scientific, Wilmington, USA). The DNA was then stored at −20 °C until further analysis.

### PCR array analysis

The PCR array was conducted using the WCGENE array Kit (wc-DNABAL230517, WcGene Biotech, Shanghai, China) (as detailed in Table 1) and with specific primer sequences listed in S1 Table. The quantitative PCR (qPCR) was executed using the QuantStudio 5 Real-Time PCR System from Applied Biosystems. For each reaction, a 10 μl qPCR mix was prepared, consisting of 5 μl iTaq Universal SYBR Green Supermix (Bio-Rad, Shanghai, China), 1 μl of the DNA template, 1 μl of each primer, and 2 μl of ddH_2_O. The thermal cycling conditions were initiated with a 10-minute denaturation at 95 °C, followed by 40 cycles of denaturation at 95 °C for 30 seconds and annealing at 60 °C for 30 seconds. A melting curve analysis from 60 to 95 °C followed to assess amplification specificity.

**Table 1:** List of drug resistance gene.

Item	Type	Name									
1	Aminoglycoside resistance genes	aac	aac(6’)I1	aac(6’)-Ib(aka aacA4)-01	aac(6’)-Ib(aka aacA4)-02	aac(6’)-Ib(aka aacA4)-03	aac(6’)-II	aac(6’)-Iy	aacA/aphD	aacC	aacC1
2	Beta-lactamas resistance genes	bla1	bla-ACC-1	blaCMY	blaCMY2-01	blaCMY2-02	blaCTX-M-01	blaCTX-M-02	blaCTX-M-03	blaCTX-M-04	blaCTX-M-05
		blaCTX-M-06	blaGES	blaIMP-01	blaIMP-02	bla-L1	blaMOX/blaCMY	blaOCH	blaOKP	blaOXA1/blaOXA30	blaOXA10-01
		blaOXA10-02	blaOXY	NDM1							
3	FCA resistance genes	acrA-01	acrA-02	cmlA1-01	cmlA1-02	pmrA	qnrA				
4	MGE	intI	intI1	IS613	tnpA-01	tnpA-02	tnpA-03	tnpA-04	tnpA-05	tnpA-07	Tp614
5	MLSB resistance genes	carB	ereA	erm(34)	erm(35)	erm(36)	ermA	ermA/ermTR	ermB	ermC	ermF
		ermJ/ermD	ermK-01	ermK-02	ermT-01						
6	Sulfonamide resistance genes	sul1	sul2	sulA/folP-01	sulA/folP-02	sulA/folP-03					
7	Tetracyline resistance genes	tet(32)	tet(34)	tet(35)	tet(36)-01	tet(36)-02	tet(37)	tet(38)	tetA-01	tetA-02	tetB-01
8	Vancomycin resistance genes	vanA	vanB-01	vanB-02	vanC-01	vanC-02	vanC-03	vanC1	vanC2/vanC3	vanD	vanG
9	Other genes	dfrA1	dfrA12	ereB	folA						

Gene expression levels were normalized against the geometric mean of the housekeeping gene 16 SrRNA. Relative gene abundances were computed using the comparative CT method (Livak and Schmittgen, 2001). The qPCR was performed under conditions that ensured amplification efficiency was maintained between 90% and 110%. Average CT values for both ARGs and the 16S rRNA genes were calculated using: ΔCT = CT_ARG - CT_16S and ΔΔCT = ΔCT Target - ΔCT_Ref. The fold changes (FC) in gene expression were derived using: FC = 2^−ΔΔCT^, enabling comparisons of relative gene abundances across different samples.

### 16S rDNA sequencing for microbial community profiling

Total DNA was quality-controlled using a Thermo NanoDrop 2000 UV-Vis spectrophotometer and 1% agarose gel electrophoresis. The target region for 16S rDNA amplification was the V3-V4 region, using universal primers 341F and 806R. Index and adaptor sequences suitable for Illumina NovaSeq PE250 sequencing were added to the 5’ end of the universal primers, followed by the design of the specific primers: Forward Primer (5’–3’): CCTACGGGRSGCAGCAG (341F); Reverse Primer (5’–3’): GGACTACVVGGGTATCTAATC (806R). The diluted genomic DNA was used as a template for PCR amplification with the KAPA HiFi Hotstart ReadyMix PCR Kit to ensure high accuracy and efficiency of amplification. PCR products were checked using 2% agarose gel electrophoresis, and the desired fragments were recovered using the AxyPrep DNA Gel Recovery Kit (AXYGEN, USA). After recovery, library quality was assessed using the Thermo NanoDrop 2000 UV-Vis spectrophotometer and 2% agarose gel electrophoresis. Upon successful quality control, the libraries were quantified using a Qubit fluorometer. The libraries from different samples were mixed in appropriate ratios according to the required sequencing data volume. Sequencing was performed on the Illumina NovaSeq PE250 platform (Shanghai Realbio Technology Co., Ltd., China). Subsequent bioinformatics analysis was conducted to process the sequencing data.

### Statistical analysis

Using the TBtools package, a heatmap was generated to illustrate the relative abundance of each gene across samples A and B. For comparisons between two sample groups, the wilcox.test function in R was applied, while for comparisons involving more than two groups, the kruskal.test function was used. A *P*-value threshold of less than 0.05 was considered indicative of a significant difference. To assess the similarity in ARG profiles between samples A and B, Non-metric Multidimensional Scaling (NMDS) was performed using the Bray–Curtis distance matrix based on the relative abundances of ARGs. Additionally, the significance of changes in the abundance of ARGs and 16S rRNA genes across samples was evaluated using the Student’s *t*-test and one-way ANOVA, with a 95% confidence interval (CI) indicating statistical significance. All statistical analyses were conducted using SPSS 24.0 (IBM SPSS Statistics, Chicago, IL, United States).

## Results

### Analysis of microbial antibiotic resistance genes in fresh and composted pig manure

**Fig 1:**
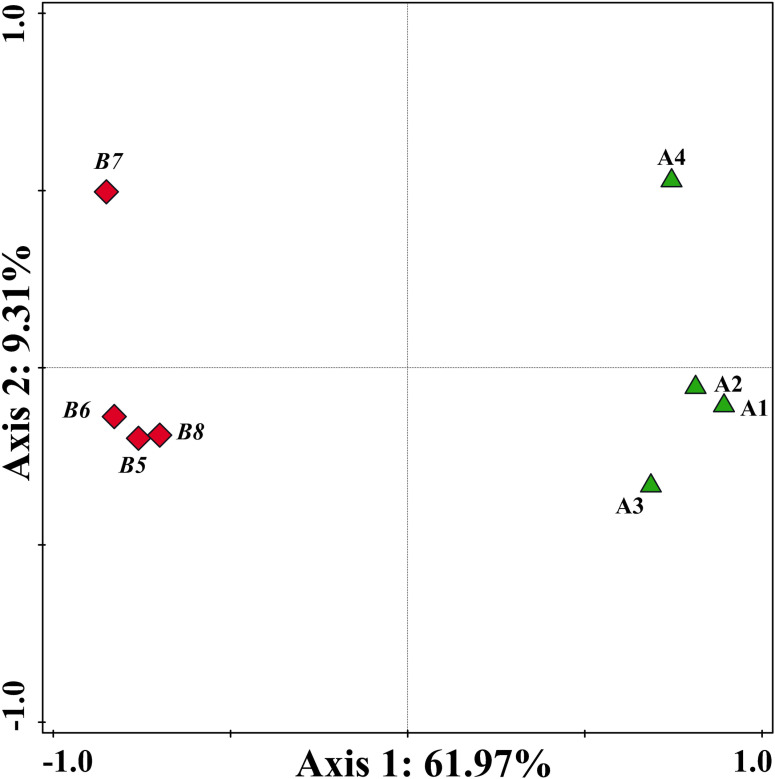
The principal coordinates analysis (PCoA) of different pig fecal samples including A1, A2, A3, A4, B5, B6, B7, and B8. A1–A4 represent fresh pig manure samples (green), and B5–B8 represent composted pig manure samples (red).

To investigate the differential expression of antibiotic resistance genes in fresh and composted pig manure, we analyzed eight samples: four from fresh pig manure (A1, A2, A3, A4) and four from composted manure (B5, B6, B7, B8) using a PCR array. Principal Coordinates Analysis (PCoA) effectively distinguished between the fresh manure samples (Group A) and the composted samples (Group B), with each group clustering distinctly as shown in Fig 1 and S2 Table. Variations in feeding practices and digestibility can influence the dispersion of data within the fresh manure group, while the extent of composting fermentation may similarly affect the composted samples. Additionally, variations in the gene amplification process during PCR analysis might contribute to data discrepancies between the groups. Despite these factors, the PCoA results clearly demonstrate significant differences between the fresh and composted manure samples, with minor variations within each group (A and B). This indicates a robust change in the microbial community and ARG profiles due to the composting process.

### Differential expression of antibiotic resistance genes in composted versus fresh pig manure

**Fig 2:**
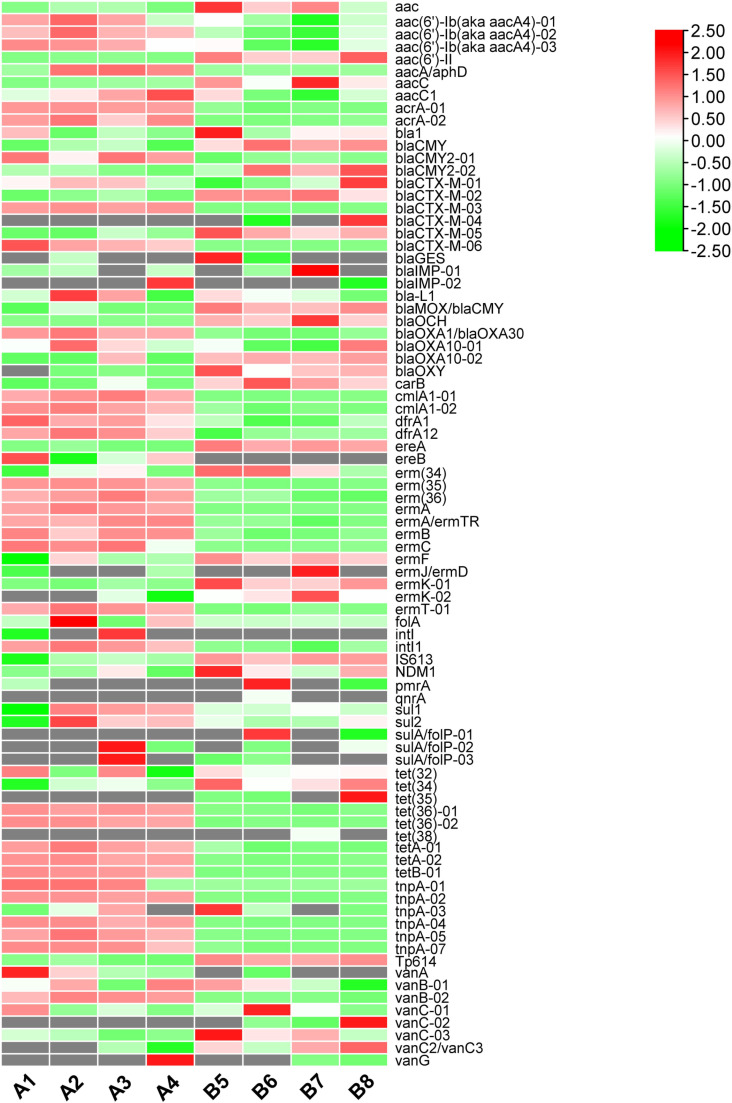
Expression heatmaps of antibiotic resistance genes (ARGs) in different pig fecal samples including A1, A2, A3, A4, B5, B6, B7, and B8. A1–A4 represent fresh pig manure samples, and B5–B8 represent composted pig manure samples.

Fig 1 analysis highlighted distinct expression levels of ARGs between fresh and composted pig manure. To delve deeper, we analyzed 85 specific ARGs, with results presented in Fig 2 and S2 Table. This analysis indicated a prevalent downregulation of ARGs in composted manure. Notably, genes associated with aminoglycoside resistance such as *aac(6’)-Ib(aka aacA4)-01*/*02*/*03* and *acrA-01*/*02*, beta-lactamase genes like *blaCTX-M-03* and *blaOXA1*/*blaOXA30*, chloramphenicol resistance genes (*cmlA1-01*/*02*), macrolide-lincosamide-streptogramin B resistance genes including *erm(34)/(35)/(36)/A/B/C/T-01*, mobile genetic elements (MGEs) such as *intI1* and *tnpA-01/02/04/05/07*, and tetracycline resistance genes like *tet(36)-01/02*, *tetA-01/02*, and *tetB-01* were significantly reduced post-composting. Conversely, certain ARGs exhibited an upregulation in composted manure, including beta-lactamase genes such as *blaCMY* and *blaCTX-M-02*, MLSB resistance genes like *carB*, *ereA*, and *ermK-01*, and MGEs including *IS613* and *Tp614*, as well as tetracycline resistance genes like *tet(34)*. These findings underscore the substantial impact of composting on ARG dynamics, demonstrating not only a reduction in the abundance of many critical ARGs but also an increase in others. This complexity suggests that while composting generally mitigates ARG prevalence, it can also selectively enhance the expression of certain resistance genes, depending on the environmental and microbial conditions within the compost. Further research is essential to fully understand the mechanisms governing ARG modulation during the composting process and to optimize this method for maximum reduction of ARGs in agricultural settings.

### Reduced antibiotic resistance gene expression in composted compared to fresh pig manure

**Fig 3:**
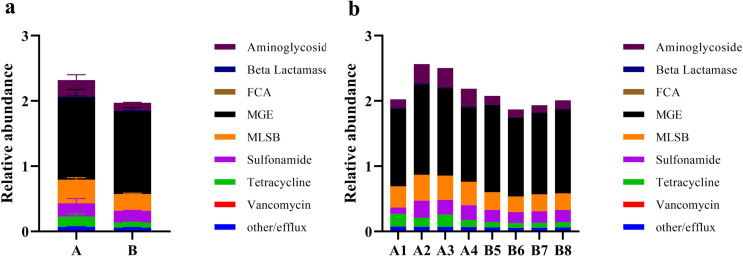
Relative abundance of antibiotic resistance genes (ARGs) in fresh and composted pig manure. The stacked histograms depict changes in ARG abundance across the samples. Different colors indicate various antibiotic types, with ARG abundance quantified as the number of ARG copies per copy of the 16S rRNA gene. Panel (a) shows aggregate data: A for all fresh pig manure samples, and B for all composted pig manure samples. Panel (b) presents individual data: A1–A4 for each fresh pig manure sample, and B5-B8 for each composted pig manure sample.

To determine whether ARGs associated with processes such as Aminoglycoside, Beta Lactamase, FCA, MGE, MLSB, Sulfonamide, and Tetracycline were differentially expressed, we conducted high-throughput qPCR and subsequent expression analysis based on gene expression abundance (Fig 3 and S3 Table). The results showed that in the composted pig manure samples B5, B6, B7, and B8 (collectively referred to as group B), the expression levels of ARGs involved in processes like Aminoglycoside, MGE, MLSB, Sulfonamide, and Tetracycline were significantly lower than in the fresh pig manure samples A1, A2, A3, and A4 (collectively referred to as group A). This indicates that the expression of ARGs in these antibiotic processes is consistently lower in composted pig manure compared to fresh pig manure.

### The differentially expressed antibiotic resistance genes between composted pig manure and fresh pig manure

**Fig 4:**
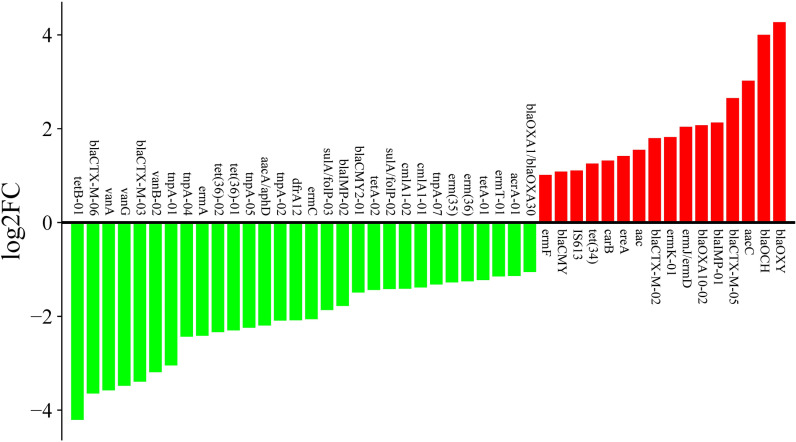
Fold change of antibiotic resistance genes (ARGs) between A and B of the pig fecal samples. Red represents upregulated genes, and green represents downregulated genes. FC, fold change.

To further screen for differentially expressed antibiotic resistance genes in composted pig manure and fresh pig manure, we calculated the relative expression abundance based on the log2FC formula. We found that compared to fresh pig manure, 30 ARGs including *tetB-01*, *blaCTX-M-06*, *vanA*, *vanG*, *blaCTX-M-03*, *vanB-02*, *tnpA-01*, *tnpA-04*, *ermA*, *tet(36)-02*, *tet(36)-01*,*tnpA-05*, *aacA/aphD*, *tnpA-02*, *dfrA12*, *ermC*, *sulA/folP-03*, *blaIMP-02*, *blaCMY2-01*, *tetA-02*, *sulA/folP-02*, *cmlA1-02*, *cmlA1-01*, *tnpA-07*, *erm(35)*, *erm(36)*, *tetA-01*, *ermT-01*, *acrA-01*, and *blaOXA1/blaOXA30*, were downregulated in composted pig manure, while 19 ARGs, including *ermF*, *blaCMY*, *IS613*, *tet(34)*, *carB*, *ereA*, *aac*, *blaCTX-M-02*, *ermK-01*, *ermJ/ermD*, *blaOXA10-02*, *blaIMP-01*, *blaCTX-M-05*, *aacC*, *blaOCH* and *blaOXY*, were upregulated (Fig 4 and S4 Table). These results indicate that the expression levels of ARGs produced in composted pig manure are significantly different from those in fresh pig manure.

### The screen of differentially expressed antibiotic resistance genes

**Fig 5:**
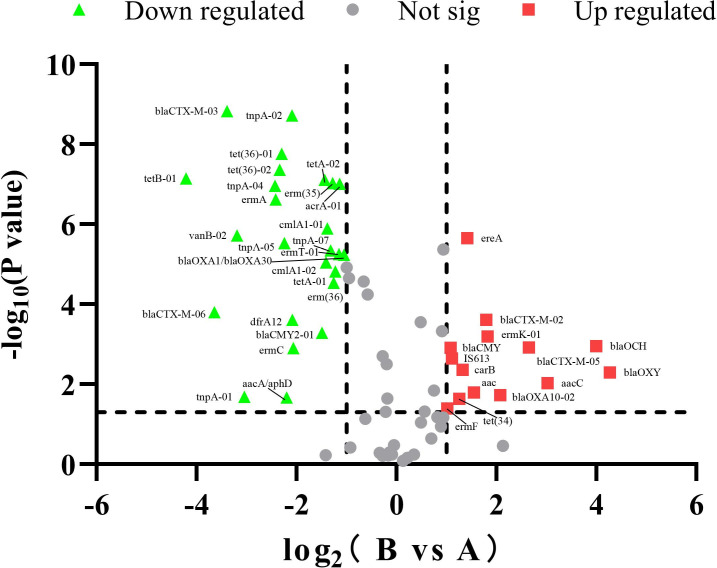
The fold change and P value distribution of antibiotic resistance genes (ARGs) in B (composted pig manure) vs A (fresh pig manure).

To explore the differential expression of antibiotic resistance genes between composted and fresh pig manure, we analyzed the fold change and P value distribution of these genes. Utilizing criteria of log2FC > 1 and *p* < 0.05, we identified 39 differentially expressed ARGs between the A and B samples, including 14 upregulated and 25 downregulated ARGs (Fig 5 and S5 Table).

### Analysis of antibiotic resistance gene distribution in various samples

**Fig 6:**
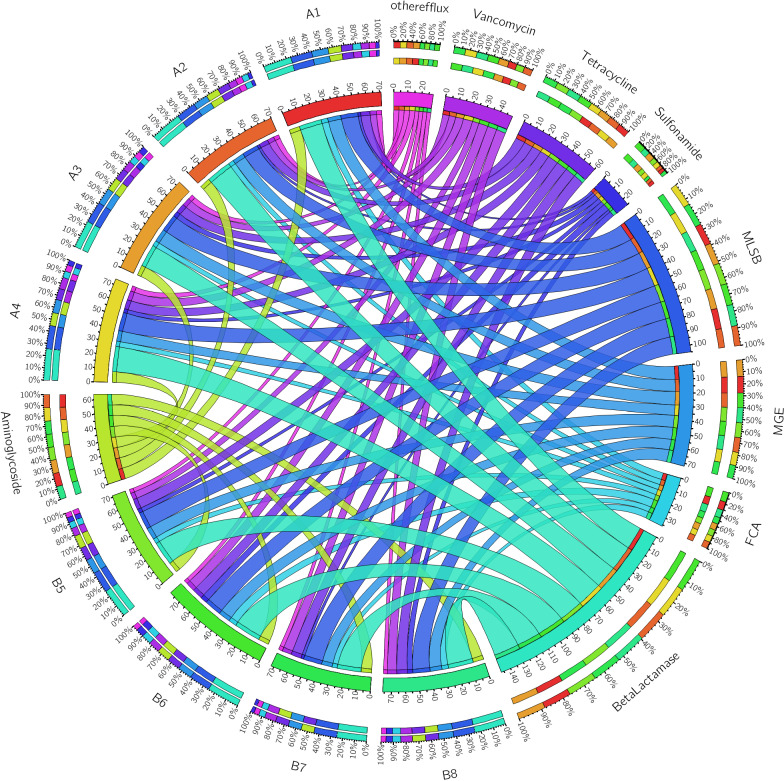
The number and percentage of different antibiotic resistance genes in different samples including fresh pig manure A1, A2, A3, and A4, and composted pig manure B5, B6, B7, and B8.

To elucidate the distribution of each type of ARG across different samples, we utilized circular graphs to depict both the number and percentage of ARGs. We observed that ARGs associated with Aminoglycoside, Beta-Lactamase, FCA, MGE, MLSB, Sulfonamide, Tetracycline, and Vancomycin were present in all samples. Notably, Tetracycline resistance genes were more prevalent in composted pig manure than in fresh pig manure, both in terms of number and proportion (Fig 6 and S6 Table). This suggests a distinctive persistence or accumulation of Tetracycline resistance genes in the composted samples.

### Comparative analysis of microbial community structure in fresh and composted pig manure

**Fig 7:**
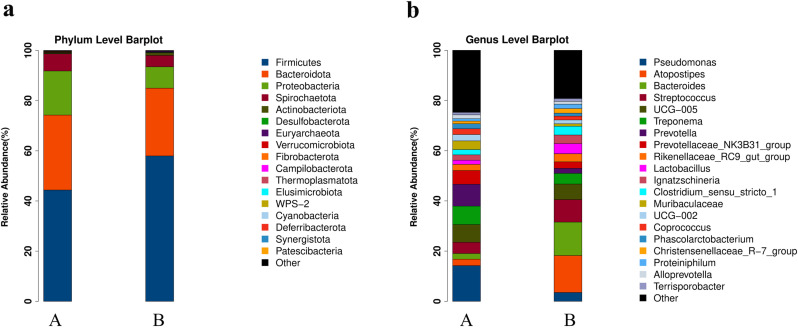
The bacterial community composition at the phylum level (a) and genus level (b) in different samples including fresh pig manure A and composted pig manure B.

We performed species profiling using 16S rRNA sequencing data at both the phylum and genus levels for two distinct sample groups: fresh pig manure (Group A) and composted pig manure (Group B). Results are visualized in bar charts that illustrate the relative abundance of microbial taxa within each group. At the phylum level, both fresh and composted pig manure samples demonstrated a rich diversity in microbial community composition. In Group A, the most abundant phyla were Firmicutes and Bacteroidetes, with lesser quantities of Proteobacteria, Actinobacteria, and Fusobacteria. In contrast, the microbial community in Group B showed a notable shift characterized by a significant increase in Firmicutes and a marked reduction in Bacteroidetes. The prevalence of other phyla such as Proteobacteria and Actinobacteria remained relatively unchanged, whereas Fusobacteria notably declined in the composted samples (Fig 7a and S7 Table).

At the genus level, Clostridia, a class within Firmicutes, predominated in both sample groups, though it was more abundant in the composted manure (Group B). Fresh pig manure (Group A) exhibited a higher relative abundance of Bacteroidia (within Bacteroidetes), whereas the presence of Betaproteobacteria diminished in the composted group (Fig 7b and S7 Table). The species profiling of 16S rRNA highlighted considerable shifts in the microbial community structures between fresh and composted pig manure. The composting process notably influenced the microbial phyla and genera distribution, with an observed increase in Firmicutes and a decrease in Bacteroidetes in the composted samples. These alterations suggest that composting significantly impacts the microbial community composition, potentially driven by environmental changes such as temperature and moisture during the process. The results indicate that composting could promote the proliferation of certain microbial populations while suppressing others, potentially affecting the persistence of ARGs and the overall efficacy of manure management.

## Discussion

Livestock and poultry farming are essential to agriculture and play a critical role in improving the quality of life for populations worldwide [30]. However, intensified farming often involves the use of antibiotics in animal feed to promote growth and enhance immunity [31]. Despite the implementation of the “Animal Drug Management Regulations” by the Chinese government in 2004, a significant amount of antibiotics—over 84,000 tons, representing 52% of total antibiotic usage—was still being administered to animals by 2013 [32]. This leniency in antibiotic regulation has led to the widespread presence of antibiotic residues in animal feces, with concentrations often exceeding hundreds of milligrams of tetracycline per kilogram [33,34]. The introduction of antibiotics into modern medicine and agriculture marked a transformative era that began with Alexander Fleming’s discovery of penicillin in 1928 [35]. Initially celebrated for their therapeutic potential, antibiotics soon became ubiquitous as growth promoters in food production [36]. However, their pervasive use in clinical settings, agriculture, and the food industry has precipitated the rise of multi-drug-resistant bacteria, presenting a formidable challenge to public health [37].

The overuse and misuse of antibiotics in livestock farming have escalated concerns about the emergence and spread of antibiotic resistance genes (ARGs), now recognized as emerging pollutants with significant implications for global health. Intensive livestock operations are key contributors to the environmental load of ARGs [23]. In this study, we employed high-throughput quantitative PCR arrays to analyze ARG profiles in both composted and fresh pig manure. Our analysis revealed significant differences in microbial antibiotic resistance between the two manure types (Fig 1), with a total of 39 differentially expressed ARGs identified (Fig 5). Our findings demonstrate high levels of antibiotic residues in pig manure, highlighting the substantial role of livestock farming in contributing to environmental antibiotic pollution. The detection of diverse ARGs in both fresh and composted manure samples underscores the urgent need to understand the dynamics of antibiotic resistance in agricultural settings.

Composting is a prevalent method for managing organic waste, including livestock manure, yet its influence on antibiotic resistance dynamics remains insufficiently explored. Animal manure is a significant reservoir for ARGs, particularly those conferring resistance to commonly used livestock antibiotics such as tetracyclines, sulfonamides, and fluoroquinolones, posing substantial public health risks. The dynamics of ARGs during composting have been highlighted in various studies. For instance, a PCR analysis of 83 human fecal samples from three different tribal populations indicated a predominance of tetracycline resistance genes, with a similar distribution of 35 ARGs across the groups [23]. Additionally, investigations into pig manure from various farms identified 149 unique ARGs [38]. Liu et al. (2020) utilized a high-throughput quantitative PCR array to detect ARGs and mobile genetic elements in antibiotic-free chicken farms, which are known to facilitate the spread of ARGs [39]. Composting, a dynamic process composed of mesophilic, thermophilic, cooling, and maturing stages, is characterized by significant temperature fluctuations resulting from microbial activity. This treatment has been shown to substantially decrease the overall expression levels of ARGs, especially tetracycline resistance genes such as *tetM*, *tetW*, *tetO*, and *tetS*, which decline progressively through the composting stages [14]. Our study builds upon these findings by providing detailed insights into the effects of composting on ARG expression in pig manure. We observed notable differences in ARG expression between fresh and composted pig manure samples, suggesting that composting influences the composition and abundance of antibiotic-resistance traits in microbial communities. A comparative analysis identified 39 differentially expressed ARGs, with *tetB-01*, *blaOCH*, and *blaOXY* being the most significantly upregulated in composted manure (Fig 5). Additionally, our results revealed a marked reduction in ARGs associated with Aminoglycoside, MLSB, Sulfonamide, and Tetracycline in composted manure compared to fresh manure (Fig 3). This decrease aligns with previous studies that have quantified ARGs in various manure samples from poultry, swine, and pigs, underscoring the potential of composting as an effective management strategy to mitigate ARG prevalence in agricultural settings [40–42].

These findings underscore the dynamic nature of antibiotic resistance, influenced by environmental factors such as composting. Gaining an understanding of the factors that drive ARG dynamics during composting is essential for formulating effective strategies to curtail the spread of antibiotic resistance in agricultural settings. Identifying specific ARGs that are differentially expressed during composting offers critical targets for surveillance and control measures. Monitoring these genes enables policymakers and agricultural practitioners to evaluate the efficacy of waste management practices in mitigating the dissemination of antibiotic resistance. Moreover, the impact of seasonal variations on microbial communities is profound. For instance, the favorable conditions during summer facilitate the rapid proliferation of dominant microbial communities, which can suppress the growth of other microorganisms. Conversely, in winter, the colder temperatures allow bacteria to survive longer and grow more slowly, leading to the coexistence of diverse bacterial types [43,44]. This seasonal variation can lead to mixed infections involving multiple bacteria, increasing the complexity of diseases and the diversity of ARGs.

This study highlights the effectiveness of composting in reducing ARGs in animal manure. However, to offer a broader perspective, it is crucial to compare composting with other manure management practices, such as anaerobic digestion (AD). Both methods effectively reduce ARG abundance, but their mechanisms differ significantly. Composting relies on aerobic microbial activity and elevated temperatures, whereas AD operates under anaerobic conditions and involves biogas production processes. Notably, AD is particularly effective in reducing certain ARGs, such as those associated with sulfonamide resistance, especially under thermophilic conditions where high temperatures disrupt ARG-carrying microbial communities [45,46]. However, AD may be less effective against tetracycline resistance genes under mesophilic conditions. These distinctions underscore the importance of selecting manure management practices based on specific ARG profiles and environmental conditions. The applicability of these findings extends across various livestock species and geographic regions due to the universal microbial processes involved in composting. However, factors such as diet and antibiotic use significantly influence the composition of manure, affecting the persistence and reduction rates of ARGs. Future research should evaluate the effectiveness of composting in different settings, including manure from poultry, cattle, and other livestock, to assess its generalizability [47,48].

Despite its benefits, residual ARGs in composted manure could still pose risks when applied to agricultural fields, potentially spreading to soil, water, and crops. This dissemination facilitates the horizontal transfer of ARGs to pathogenic bacteria, exacerbating challenges in managing antibiotic resistance in both human and animal populations. Future studies should explore the persistence and mobility of ARGs in diverse agricultural environments. Comparative analyses of composting and other strategies like anaerobic digestion will provide essential insights for optimizing manure management practices to minimize ARG dissemination and protect public health [45,46]. While this study offers valuable insights into the impact of composting on antibiotic resistance in pig manure, further research is necessary to elucidate the underlying mechanisms that drive ARG dynamics during composting. Investigating the long-term effects of compost application on soil microbial communities and ARG persistence would yield crucial data for sustainable agricultural practices. Additionally, comparative studies across different livestock species and geographic regions could enhance our understanding of the broader implications of antibiotic use in agriculture [38,40–42]. Our findings demonstrate significant differences in ARG expression levels between fresh and composted pig manure, indicating the potential impact of composting on antibiotic resistance dynamics. These results emphasize the importance of implementing sustainable waste management practices in livestock farming to mitigate the spread of antibiotic resistance in the environment. Addressing these challenges is vital for preserving both human health and environmental integrity amid growing antibiotic resistance threats. One limitation of this study is the small sample size and the restricted range of fecal samples from different composting durations. Another constraint is the absence of environmental factor measurements, which precludes quantifying their impact on ARGs. Future research should investigate various types of farms and environmental conditions to further our understanding and improve farm management practices to reduce ARG pollution.

## Conclusions

This study identified significant differences in the expression levels of antibiotic resistance genes (ARGs) between fresh and composted pig manure, with most ARGs being downregulated during composting. Additionally, 16S rRNA gene sequencing revealed that the composting process significantly reshaped the microbial community structure, highlighting its potential role in altering both ARG expression and microbial composition in pig manure.

## Supporting information

S1 Table Primers sequences of drug resistance genes. (XLSX)

S2 Table Serves as the data source for Figs 1 and 2. (XLSX)

S3 Table Serves as the data source for Fig 3. (XLSX)

S4 Table Serves as the data source for Fig 4. (XLSX)

S5 Table Serves as the data source for Fig 5. (XLSX)

S6 Table Serves as the data source for Fig 6. (XLSX)

S7 Table Serves as the data source for Fig 7. (XLSX)
